# The Necessity of Routine Pre-operative Group and Save Testing for Emergency Appendectomies

**DOI:** 10.7759/cureus.50032

**Published:** 2023-12-06

**Authors:** Basil Ibrahim, Emmanuel Obayi, Mohammed Barghash, Usifoh Itaman, Yazan Alkurdi, Anna Johnson, Moustafa Mansour

**Affiliations:** 1 Surgery, Manchester Foundation Trust, Manchester, GBR; 2 General Surgery, North Manchester General Hospital, Manchester, GBR; 3 Upper Gastrointestinal Surgery, North Manchester General Hospital, Manchester, GBR; 4 Upper GI Surgery, North Manchester General Hospital, Manchester, GBR

**Keywords:** pre-operative planning, blood loss in appendicectomy, emergency appendicectomy, blood group and save, pre-operative workup

## Abstract

Background

Pre-surgical group and save (G&S) tests are routine but can result in unnecessary expense and theatre delays. The objective of this study was to assess the necessity of G&S testing prior to appendectomy and evaluate the cost implications.

Methods

This retrospective study analysed the records of 200 patients with appendicitis who underwent emergency appendectomies at a busy general surgery department between March 2021 to August 2022. The study adhered to local clinical governance unit protocol and the Strengthening the Reporting of Cohort Studies in Surgery (STROCCS) guidelines. Patients who had elective appendectomies or other emergency procedures were excluded. Data was collected on age, gender, number of samples and requirement for perioperative transfusion. Comparisons were drawn between patients who underwent laparoscopic, open or converted emergency appendectomies.

Results

Of the sample population, (median age 32, 55.5% male), 93.5% had valid preoperative G&S tests. None required perioperative blood transfusions. 26% of the patients only required one sample for a valid G&S due to having previous sample in the lab; 55% required two samples; 7% needed a third sample because one initial sample was rejected; and 5.5% required four samples because the initial two samples were rejected. The total cost of these samples was estimated to be £3,500.14.

Conclusion

Emergency appendectomy poses minimal risk of resulting in the need for blood transfusions. Reevaluating the need for routine preoperative G&S testing and adopting a risk-benefit analysis approach could have a financial benefit for the NHS.

## Introduction

Blood grouping and crossmatching, commonly referred to as group and save (G&S) testing, is a crucial medical procedure that involves determining the ABO and RhD blood groups of a patient, while also screening for the presence of any atypical antibodies. The information obtained from this test is vital in ensuring that medical staff provide the appropriate blood transfusion to patients when required, thereby preventing the occurrence of any adverse reactions or complications [1]. Previous research has indicated that the rates of blood transfusion during laparoscopic general surgery are generally low, with transfusion primarily occurring because of complications or specific risk factors [2]. The objective of this study was to determine if routine blood grouping and crossmatching prior to laparoscopic and open appendectomy procedures is necessary or if the approach could be altered for cost and time efficiency.

Laparoscopic surgery is a minimally invasive surgical technique that has gained widespread popularity in recent years. Appendectomies are one of the most emergency surgical procedures performed in hospitals with the laparoscopic approach being the most used. While it is generally considered to be a safe and effective procedure, there is a small risk of major vascular injury during the surgery [3-5]. Intraoperative bleeding is a relatively uncommon occurrence, but when it does occur, it can be life-threatening and require prompt intervention. Prompt and appropriate management of these injuries can be achieved with the right resources. However, in rare cases where the use of group-specific blood products may be detrimental, alternative measures are required. The insertion of trocars during laparoscopy may cause major vessel injury, necessitating administration of blood products. It is worth noting that historically, catastrophic hemorrhage has been primarily attributed to injuries sustained by the aorta, vena cava, and internal iliac vessels [3].

It is essential that healthcare providers are aware of the small but significant risk of major vascular injuries during laparoscopic surgery. By ensuring that essential resources such as O-negative blood, platelets, cryoprecipitate, fresh frozen plasma, and tranexamic acid are readily available, healthcare providers can ensure the prompt and appropriate management of these injuries, thus reducing the risk of adverse outcomes for their patients. Hemorrhage may also result from direct trauma to vessels of the anterior abdominal wall such as the inferior epigastric arteries [4,5]. While any instance of injury during laparoscopic surgery is concerning, it's worth noting that the frequency of bowel and major vessel injuries is quite low, occurring in only 0.04% and 0.02-0.04% of cases, respectively [6]. In general, appendectomy is a safe routine emergency operation with minimal blood loss, with most procedures recorded as none or less than 500 millilitres in the operative notes [7].

There are currently no established national guidelines in the UK for blood group and antibody screening of patients who require emergency laparoscopic general surgery, such as an appendectomy [2]. The latest guidelines from the National Institute for Health and Care Excellence on preoperative tests for elective surgery, which were published in 2016, do not recommend routine G&S tests, even for major or complex surgical procedures [8]. This implies that G&S tests are not considered to be a necessary component of preoperative testing and are not required to be conducted on a routine basis for all elective surgical procedures, irrespective of their complexity or severity. This recommendation is in line with current best practices and serves to streamline the preoperative testing process while maintaining patient safety and reducing unnecessary healthcare costs. There were no recommendations for emergency surgery [8]. The French Society of Anaesthesia and Intensive Care recommends against the routine use of blood grouping and crossmatching in emergency laparoscopic procedures if the bleeding risk is low, according to guideline issued in 2012 [9]. Furthermore, several studies have been undertaken to evaluate the necessity of routine blood grouping and crossmatching in laparoscopic general surgery [1,2,10]. While these studies have certain limitations, some indicate that blood grouping and crossmatching should be given due consideration in emergency laparoscopic procedures, particularly for patients with established risk factors, such as a history of cardiovascular co-morbidities, coagulopathy, anemia, or hematological malignancy [10]. There is a lack of studies assessing the need for blood transfusion in open appendectomies.

## Materials and methods

Participants, interventions, comparisons, outcomes (PICO) research question

Is routine G&S testing cost-effective for patients undergoing emergency appendectomy?

Study design and patient selection

We conducted a retrospective cross-sectional study following a predefined protocol recommended by the local Clinical Governance Unit. The study was reported in compliance with The Strengthening the Reporting of Cohort Studies in Surgery (STROCSS) guideline for observational studies [11]. There was no need for Research Ethics Committees approval and patient consent as the study was retrospective and the data used were unidentifiable. The study was conducted in the General Surgery Department of North Manchester General Hospital in the United Kingdom (UK).

Patients who underwent emergency appendectomy over a period of 18 months between March 2021 and August 2022 were included. Patient details were identified and extracted from the hospital maintained electronic health records. Patients who had other emergency laparoscopic or open procedures and those who had elective or interval appendectomies were excluded.

Comparisons

We aimed to synthesize outcomes for the following: Emergency laparoscopic appendectomy, emergency open appendectomy, and emergency laparoscopic converted to open appendectomy.

Outcomes

The outcome of this study was to assess the appropriateness and necessity of routine G&S testing for patients undergoing emergency appendectomy. The study also aimed to evaluate the cost implications of G&S testing on our Trust and this can be reflected on NHS as a whole.

Data collection

To ensure comprehensive data collection, a proforma was created for electronic use. This proforma was designed to collect the following data: patients’ age, gender, G&S testing, number of samples and need for blood transfusion either intra-operative or post-operative. Number of samples run by the lab and total costs were calculated.

Data synthesis and statistical analyses

The categorical variables were summarised using absolute and relative frequencies and were compared using Chi-square test. The continuous variables were summarized using mean ± standard deviation and median (inter-quartile range) and were compared using The Kruskal-Wallis test. All statistical tests were two-tailed and statistical significance was assumed at P < 0.05. The statistical analyses were performed using IBM SPSS Statistics v.25

## Results

Patient characteristics

200 patients had emergency appendectomy between March 2021 and August 2022, of which 160 patients (80%) were laparoscopic, 28 patients (14%) were open, and 12 patients (6%) had laparoscopic converted to open appendectomy. The median age of the included patients was 32 (interquartile range of 23) and 55.5% of them (111 out of 200) were male. 93.5% of patients (187 out of 200) had valid G&S tests prior to the procedure and only 6.5% of patients (13 out of 200) underwent the procedure without any G&S testing (Table 1). None of the patients in the included population needed blood transfusion in the perioperative period (Table 2).

**Table 1:** Baseline characteristics of the included population KW: Kruskal Wallis test, c2: Chi square test; G&S: Group and save

	Total (N=200)	Laparoscopic (N=160, 80%)	Open (N=28, 14%)	Laparoscopic converted to open (N=12, 6%)
Age on arrival				
Mean ± SD	33.45± 16.35	32.44 ± 14.34	32.18 ± 22.89	49.83± 16.17
Median (IQR)	32 (23)	31 (22)	26 (36)	52 (21)
KW (p)		11.657 (p = 0.003)		
Sex				
Male	111 (55.5%)	88	19	4
Female	89 (44.5%)	72	9	88
c^2 ^(p)		4.135 (p = 0.127)		
G&S				
Yes	187 (93.5%)	158	18	11
No	13 (6.5%)	2	10	1

**Table 2:** Number of samples taken, and number of samples run by the lab and total costs G&S: Group and save

	Number of samples run by the lab	Total number of patients having G&S	Laparoscopic	Open	Laparoscopic converted to open
0 sample	0	13 (6.5%)	2	10	1
1 sample	52	52 (26%)	37	7	8
2 samples	220	110 (55%)	98	10	2
3 samples, 1 rejected	28	14 (7%)	12	1	1
4 samples, 2 rejected	22	11 (5.5%)	11	0	0
Total number of samples run by the lab = 322					
Cost		£10.87 X 322 = £3,500.14			
Patients needed transfusion		0			

Number of samples taken and total costs

A total number of 52 patients (26%) needed one sample only to get a valid G&S testing because of previous valid samples in the lab. Another 100 patients (55%) needed two samples to get a valid G&S testing. 7% of patients (14 out of 200) needed a third sample because one of the two samples was rejected, and 11 patients (5.5%) needed an extra two samples because the initial two samples were rejected. Total numbers of samples run by the lab were calculated to be 322 samples for the 200 patients. The cost of a single G&S sample testing was estimated by the Hospital Transfusion Committee to be £10.87 in our trust. Therefore, running G&S testing on those 322 samples was estimated to have costed the trust about £3,500.14 (Table 2).

## Discussion

Based on the results of our retrospective study, the necessity of routine blood grouping and/or crossmatching for patients undergoing emergency laparoscopic and open appendectomy procedures may be questionable. The study reveals no patient requiring transfusion among those undergoing emergency laparoscopic and open appendectomy, suggesting that routine blood grouping and/or crossmatching may not be indispensable for this particular patient population. To further assess the results, a comprehensive review of literature was undertaken.

Fadel et al. conducted a systematic review evaluating routine preoperative G&S testing for cholecystectomy and appendectomy procedures with 15 retrospective studies, totaling 477,437 patients. The study conducted revealed that 37.8% of the cases had G&S tests prior to operation, resulting in an overall perioperative transfusion rate of 2.1%. The primary preoperative risk factors associated with transfusion were cardiovascular co-morbidity, anemia, hematological malignancy, and coagulopathy. The author reported that all 15 studies in the systematic review recommended that routine G&S testing is unnecessary for cholecystectomy and appendectomy procedures. The costs of testing per sample range from £15.00 to £21.30, with potential annual costs of £3,925.00 to £22,075.00 [10].

Ghirardo et al. performed a retrospective analysis of their database including a total of 3,424 patients who underwent routine preoperative type and screen testing before cholecystectomy, hernia repair, or appendectomy based on the risk of transfusion. The risk of transfusion was found to be 0.32%. They concluded a low probability of requiring blood products during or immediately after surgery which supports the elimination of routine testing without compromising the quality of patient care. They also estimated annual savings of $55,000 at their parent institution [12].

Likewise, a study by Farrell et al. investigated blood transfusion rates and cost-effectiveness of blood group and antibody screening during laparoscopic emergency appendectomy. The study showed a low transfusion rate for emergency laparoscopic surgeries, with a rate of 0.2% for emergency appendectomies. The study also discussed the use of blood of O-negative type for cases requiring massive transfusion in the perioperative period. It highlighted that while conventional wisdom suggests type-specific transfusions to minimize risk, transfusing O-negative type blood is associated with a minimal increased risk of non-ABO alloantibody incompatibility. They also concluded that it is highly unlikely for a patient who undergoes emergency appendectomy to require blood transfusion that is not available and estimated the chance to be (0.003%) [13].

Barrett-Lee et al. conducted a study to evaluate the frequency of blood transfusion in emergency laparoscopic general surgery cases and aimed to determine the need of routine blood group and antibody screening for them. They collected data from 562 emergency laparoscopic cases. Most patients underwent laparoscopic appendectomy, diagnostic laparoscopy, or laparoscopic cholecystectomy. Out of the total patient cohort, 91.5% had a first G&S, 70.1% had a second G&S, and 5.3% had no G&S. Only 0.71% of patients had detectable antibodies on screening, and only one patient (0.18%) had a transfusion. This patient was transfused perioperatively for chronic anemia, not due to major intraoperative hemorrhage. The authors proposed a more targeted approach to preoperative G&S and suggest using O negative type blood in the event of acute hemorrhage from major vascular injury [2].

Similarly Alyacoubi, Taj, and Raza examined the necessity of routine G&S screening prior to emergency laparoscopic surgery. Their study analyzed data from 451 patients. Results showed that out of the 930 G&S samples sent, 786 (84.5%) were tested, with the rest not processed for different reasons. Among the participants in the study, 68.3% had two G&S samples sent, while 9.1% had only one sample sent. Only two patients (0.4%) required perioperative transfusion, and the indication for transfusion was not directly related to the operation. The cost analysis showed that processing a single G&S sample costs £12, and if each patient in the study cohort had two samples processed, the total cost would have been £1,182,696 [1].

It is important to note that our study has some potential limitations. First, the retrospective nature of the study design necessitates that inherent limitations be taken into account when interpreting the findings. Second, we were unable to identify risk factors in our cohort of patients that could increase their risk of needing blood transfusion during surgery due to the retrospective nature of the study. However, considering that no patient required blood transfusion disqualifies the need to factor in the effect of these risk factors. Lastly, the study was conducted at a single center and may not be the best representative of the entire population.

## Conclusions

These results align with previous studies and have shown even lower rates of blood transfusion during both laparoscopic and open appendectomies, indicating no need for routine G&S testing for such procedures. However, it is important to note that the decision to perform G&S testing for perioperative blood transfusion can be based on individual patient characteristics and the presence of specific risk factors which was not measured in this study. Based on the results of this study, a more targeted approach to preoperative G&S testing is proposed.
